# A tensor-based bi-random walks model for protein function prediction

**DOI:** 10.1186/s12859-022-04747-2

**Published:** 2022-05-30

**Authors:** Sai Hu, Zhihong Zhang, Huijun Xiong, Meiping Jiang, Yingchun Luo, Wei Yan, Bihai Zhao

**Affiliations:** 1 College of Computer Engineering and Applied Mathematics, Changsha University, Changsha, 410022 Hunan China; 2 Department of Ultrasound, Hunan Provincial Maternal and Child Health Care Hospital, Changsha, 410008 Hunan China; 3 Hunan Provincial Key Laboratory of Industrial Internet Technology and Security, Changsha University, Changsha, 410022 Hunan China; 4 NHC Key Laboratory of Birth Defect for Research and Prevention, Hunan Provincial Maternal and Child Health Care Hospital), Changsha, 410100 Hunan China

**Keywords:** Tensor, Protein–protein interaction, Protein function, Bi-random walks

## Abstract

**Background:**

The accurate characterization of protein functions is critical to understanding life at the molecular level and has a huge impact on biomedicine and pharmaceuticals. Computationally predicting protein function has been studied in the past decades. Plagued by noise and errors in protein–protein interaction (PPI) networks, researchers have undertaken to focus on the fusion of multi-omics data in recent years. A data model that appropriately integrates network topologies with biological data and preserves their intrinsic characteristics is still a bottleneck and an aspirational goal for protein function prediction.

**Results:**

In this paper, we propose the RWRT (Random Walks with Restart on Tensor) method to accomplish protein function prediction by applying bi-random walks on the tensor. RWRT firstly constructs a functional similarity tensor by combining protein interaction networks with multi-omics data derived from domain annotation and protein complex information. After this, RWRT extends the bi-random walks algorithm from a two-dimensional matrix to the tensor for scoring functional similarity between proteins. Finally, RWRT filters out possible pretenders based on the concept of cohesiveness coefficient and annotates target proteins with functions of the remaining functional partners. Experimental results indicate that RWRT performs significantly better than the state-of-the-art methods and improves the area under the receiver-operating curve (AUROC) by no less than 18%.

**Conclusions:**

The functional similarity tensor offers us an alternative, in that it is a collection of networks sharing the same nodes; however, the edges belong to different categories or represent interactions of different nature. We demonstrate that the tensor-based random walk model can not only discover more partners with similar functions but also free from the constraints of errors in protein interaction networks effectively. We believe that the performance of function prediction depends greatly on whether we can extract and exploit proper functional similarity information on protein correlations.

**Supplementary Information:**

The online version contains supplementary material available at 10.1186/s12859-022-04747-2.

## Background

As the major components of cells, proteins play important roles in almost all cell functions. Biological functions are not conducted by a single protein but by a group of interacting proteins with the same or similar functions. Accurate annotation of protein function is fundamental to understand life at the molecular level, which has far-reaching influences for biomedicine and pharmacy. Therefore, how to accurately determine functions of unknown proteins is the most challenging problem of the post-genomic era. Unfortunately, biological experiments have been unable to meet the need for functional annotation of the growing sequence data hampered by their high costs and inherent difficulties. To solve this dilemma, a number of computational methods have been put forward, which implement functional annotation by discovering interacting proteins with known functions in biological networks. High throughput techniques provided high-quality and large-scale protein–protein interaction data and resulted in the emergence of network-based method [1–3] to predict protein functions. The graph-theoretic algorithm [4, 5] is also applied to annotate functions, due to the nature of PPI networks that can be modelled as graphs. However, incompleteness and errors contained in the PPI network, as well as the low coverage of protein interaction data in most species limit the performance of these approaches mentioned above.

Considering the diversity, systematicness and dynamics of protein functions, as well as the poor quality of PPI networks, a variety of approaches have been proposed to promote the prediction of protein function by incorporating multi-source biological information. The typical processing mode of these methods is to integrate and represent functionally associated attributes of proteins in the form of biological network, and then carry out annotation of functions of using diffusion algorithm [6], clustering algorithm [7]. Liang et al. construct the Protein Overlap Network (PON) [8] for functions annotation based on the observation that two proteins are likely to perform the same or similar functions if they share the same domain compositions. Sarker et al. propose a method named *GrAPFI* [9], which reconstructs a protein- protein network based on the network topology and protein domain information, and uses the label propagation algorithm to annotate functions for unknown proteins. Peng et al. construct three biological networks: protein interaction network, domain co-occurrence network and functional interrelationship network, and perform function prediction by using unbalanced random walk algorithm in these networks [10]. In our previous studies [11], we have designed a dynamic network model for annotation of functions by integrating PPI networks, gene expression profile and proteins domain information. Another commonly used processing way of these methods is to seek the most functional similar partners for unknown proteins based on the context of protein interaction networks. Zhang et al. [12] annotate unknown proteins with all functions of the neighbor which holds the highest domain context similarity in the PPI network. On this basis, Peng et al. optimize calculation of domain context similarity by supplementing the domain compositions of proteins themselves and propose DCS (Domain Combination Similarity) [13] method. Moreover, they design another protein function similarity measure DSCP (Domain combination Similarity in Context of Protein Complex) depending on the domain compositions of both proteins and complexes including them. Rehman et al. develop the FP (Functional Potential) [14] method to calculate the similarity between interacting proteins based on motif similarity, homology similarity and sequence similarity. Piovesan et al. propose function prediction methods named INGA [15] and INGA 2.0 [16], which integrate sequence similarity, domain architecture search and PPI networks. After comparing ligand similarity, sequence similarity and functional genomic similarity of proteins, O 'Meara et al. [17] find that ligand similarity and functional genomic similarity are complementary for protein function prediction. Stavros et al. propose a new co-expression measure MLC (Metric Learning for Co-expression) [18] instead of the Pearson correlation to assign a GO term-specific weight to each expression sample for gene function prediction. Gligorijević et al. design a novel graph convolution network-based protein function prediction method DeepFRI [19], which extracts sequence features from protein language models and protein structures.

These methods attempt to improve quality of PPI networks by assigning different weights to the edges at different levels corresponding to multi-omics data. They classically aggregate multiple biological data into a composite and reliable network, which tends to eliminate the topologies and attribution of the individual protein interaction networks. Research and experimental results indicate that each type of biological data has its property or correlation and can play a different role in prediction of protein functions. The way of representing different types of data source in a system with a single type of link is no longer a magic cure-all for network science. In this context, the very pressing need for protein function prediction is to find a suitable data model. Intuitively, a proper data model describing function relevance in multi-omics data should satisfy two properties: it should not only be able to describe the hierarchy and heterogeneity of biological network, but also reflect the complex relationship between multi-omics data, and it should be supported by diversified solutions and rigorous theoretical system, which is conducive to generalization to other research fields. We formalize these two properties with a multidimensional tensor model integrating the topology of PPI networks with multiple biological data and develop the RWRT (Random Walks with Restart on Tensor) method to infer protein function. The RWRT method not only improves its performance but also preserves the functional relevance between multi-omics data and their own attributes. We apply the RWRT to the yeast protein interaction network and combine it with multiple biological data, including protein complexes and domain information. Experimental results demonstrate that our proposed RWRT method outperforms six types of methods, including NC [1], Zhang [12], DCS [13], DSCP [13], PON [8] and *GrAPFI* [9].

## Methods

### Motivation

To eliminate limitations of poor quality of the underlying protein interaction data on computational approaches, researchers have concentrated on the prediction of protein function by combining PPI networks with multi-omics data. Although great progress has been made on these methods, it remains a challenge that building a suitable model to integrate network topology with biological information. The most prevalent way is to construct a single network with high confidence by weighting and summarizing PPI data and multi-omics data, which effectively eliminates the negative effects of network incompleteness. Unfortunately, it also amplifies the functional associations between proteins and introduces a lot of false functional similarity partners, which restrict the performance improvement of prediction algorithms. The conclusion stems from analysis of yeast networks, in which more than 68% of proteins are functionally associated with other proteins from a single interaction, such as physical interaction, sharing domain context, etc. Less than 1.7% of proteins are connected by their functionally similar partner through all the given biological data. The process of simply encoding multiple biological data into edge property of a single network leads to the discovery of more neighbors with similar functions, but it also inevitably introduces a large number of fictitious functions. Take the protein YAL024C as an example, which can be annotated by functions only from its physical interacting neighbors (YFR028C). However, neighbors with no functional similarity (YCR038C, YER155C and YLR310C) in the constructed single network are picked out to characterize the protein YAL024C. Meanwhile, the weight of multiple biological data in constructing a unique network varies from species to species, and even from different data sets within the same species. We believe that aggregating multiple biological data into a single and unique network is not the wisest choice. Therefore, we introduce the tensor model to characterize the functional correlation between multi-omics data and PPI networks.

Plagued by the small-world and scale-free characteristics of PPI networks, traditional short-path distance or Euclidean distance is not suitable for the measure of functional distances between proteins. As an alternative approach, random walk model provides us with a more refined way by using the flow of information through network connections as a means to establish relationships between nodes [20]. A large number of random walk-based methods have been extensively used in essential proteins identification [21], tumors classification [22], protein function prediction [10], etc., which effectively verified the effectiveness of this model in biological networks. Inspired by these findings, we developed a tensor-based random walk with restart method for protein function prediction by combination of PPI network topology and multiple biological data. In addition, there is a restart probability α in our model to ensure that a seed node can iteratively move to a random neighbour with probability α or return to its original location with probability 1-α.

**Fig. 1:**
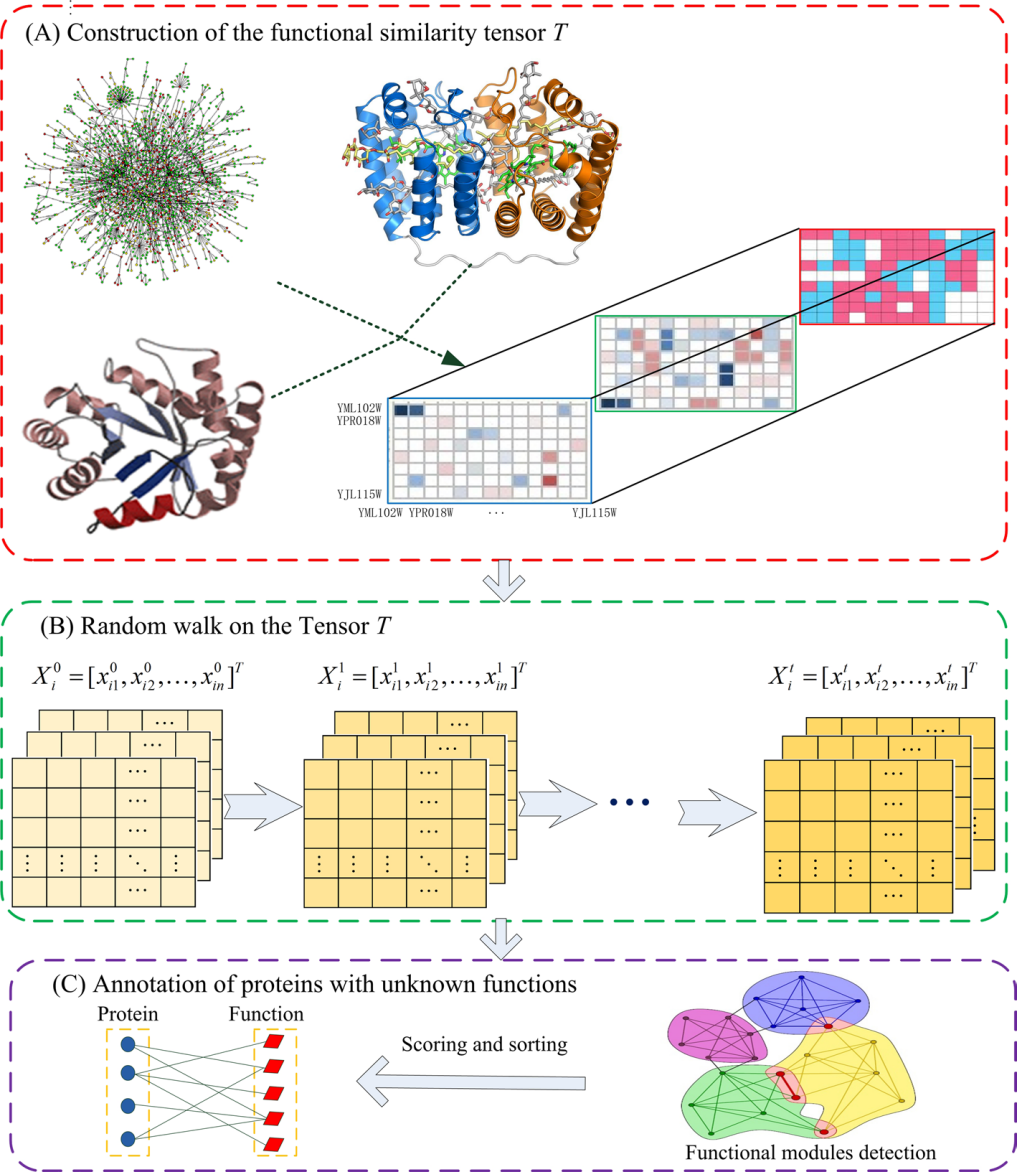
- Flowchart of RWRT method. **A** Constructing the functional similarity tensor *T* according to functional association analysis based on original PPI network, domain context and protein complex information. **B** Given a testing protein *p*_*i*_, running a bi-random walks algorithm on the tensor *T* to obtain the functional similarity vector *X*_*i*_ between *p*_*i*_ and the other proteins. **C** Removing redundant partners that have more external functional similarity than internal functional similarity within the module consisted of the target protein and its partners, and picking out top *K* of all functions of the remaining partners

Our RWRT method is composed of three major stages. First, integrating the topology of PPI networks, protein domains, and protein complexes information, a functional similarity tensor *T* is constructed. The similarity tensor preserves and reflects multiple relationships between proteins derived from multi-omics data. In the second stage, an iterative procedure calculates functional correlation score for protein pairs in the network. The iterative procedure is an extension of bi-random walks algorithm on the tensor model, which simulates a high-order Markov chain by means of two state transition tensors. In the third stage, scoring and sorting all functions of their “similar” partners (neighbors), target proteins are annotated by top *K* of these predicted functions. The flowchart for the RWRT approach is given in Fig. 1.

### Construction of the functional similarity tensor *T*

**Fig. 2:**
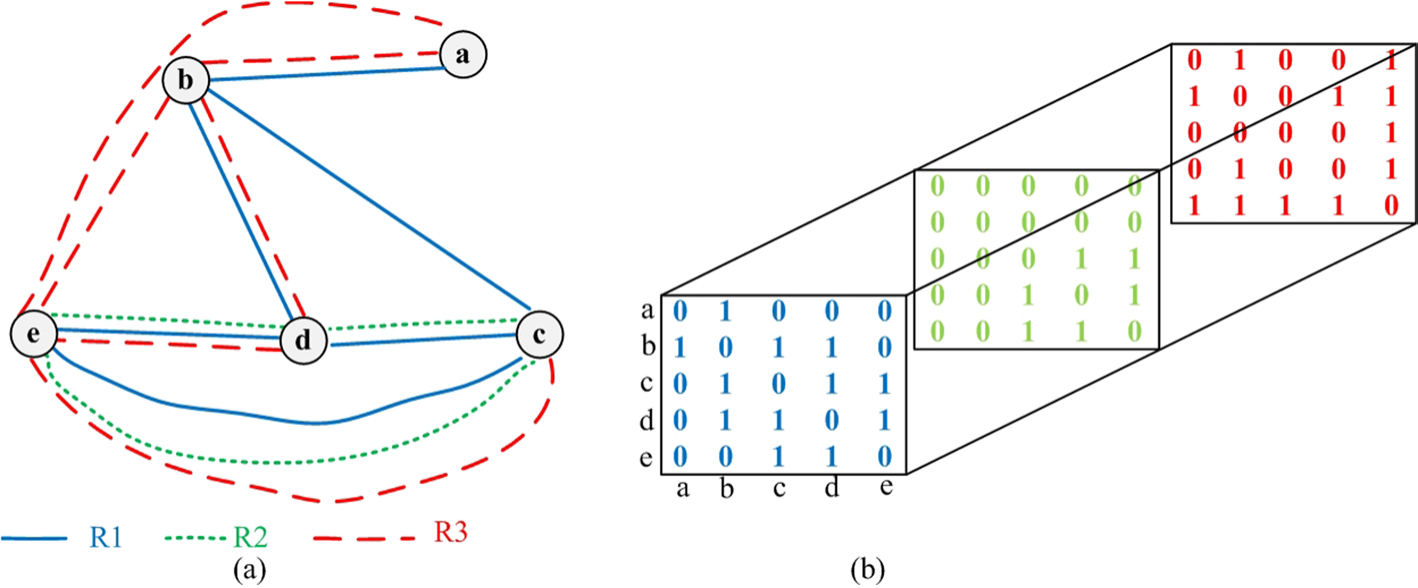
- Example of a multi-graph. **a** Showing a multi-graph consisted of five nodes and three types of connection between nodes. **b** Representing the multi-graph with a tensor model formally

The success of functional genomics is involved in the rapid accumulation of diverse biological data about genes, proteins or other macromolecules [23]. We have access to multiple types of physical or functional interactions between proteins. These different interactions with their peculiar features are better represented as a multi-graph framework. Figure 2a is an example of a multi-graph with five nodes and three types of edges. The multi-graph can also be represented as a tensor, illustrated in Fig. 2b. The tensor, as an extension of a matrix in high-order space, has many advantages for the representation and processing of complex relationships between proteins or genes [24]. For our purpose, we construct a functional similarity tensor $T \in {\mathbb{R}}^{n \times n \times m}$, where *n* and *m* represents the number of proteins and types of connection between proteins, respectively. If there is a *k*-th type of interaction between two proteins *i* and *j*, then $t(i,j,k) \in T > 0$, otherwise $t(i,j,k) \in T = 0$. In this paper, we consider three types of physical or functional association between proteins, including the physical association founded on topology of PPIs, the co-structure association derived from domain contexts and the co-module association coming from protein complex information.

The first type of association is guided by the ‘Guilt by Association’ principle. Given two proteins *p*_*i*_, *p*_*j*_ with common interacting partners, their functional similarity is estimated as follows:

1 $$t(i,j,1) = \frac{{4|N_{{p_{i} }} \cap N_{{p_{j} }} |^{2} }}{{(|N_{{p_{i} }} | + |N_{{p_{i} }} \cap N_{{p_{j} }} |) \times (|N_{{p_{j} }} | + |N_{{p_{i} }} \cap N_{{p_{j} }} |)}}$$

where $N_{{p_{i} }}$ and $N_{{p_{j} }}$ represent a set that includes *p*_*i*_ and *p*_*j*_ themselves and their direct neighbors, respectively.

Research shows that certain sets of domains are frequently found together and cooperate with each other to perform cellular functions [25]. Based on the observation, we establish the co-structure association between proteins using domain content similarity. For any two proteins *p*_*i*_ and *p*_*j*_, let *D*_*i*_ represents the set of distinct domain types in the neighbors of *p*_*i*_ with itself included, and *D*_*j*_ is that of *p*_*j*_ as well of its neighbors. *DC* denotes the set of distinct domain types contained by both neighbors of *p*_*i*_ and neighbors of *p*_*j*_, while *DT* is the set of domain types in the whole PPI networks. Then domain content similarity between *p*_*i*_ and *p*_*j*_ is calculated using the following equations:

2 $$DS(p_{i} ,p_{j} ) = - \log \frac{{\left( {\begin{array}{*{20}c} {|DT|} \\ {|DC|} \\ \end{array} } \right)\left( {\begin{array}{*{20}c} {|DT| - |DC|} \\ {|D_{i} | - |DC|} \\ \end{array} } \right)\left( {\begin{array}{*{20}c} {|DT| - |D_{i} |} \\ {|D_{j} | - |DC|} \\ \end{array} } \right)}}{{\left( {\begin{array}{*{20}c} {|DT|} \\ {|D_{i} |} \\ \end{array} } \right)\left( {\begin{array}{*{20}c} {|DT|} \\ {|D_{j} |} \\ \end{array} } \right)}}$$

**Fig. 3:**
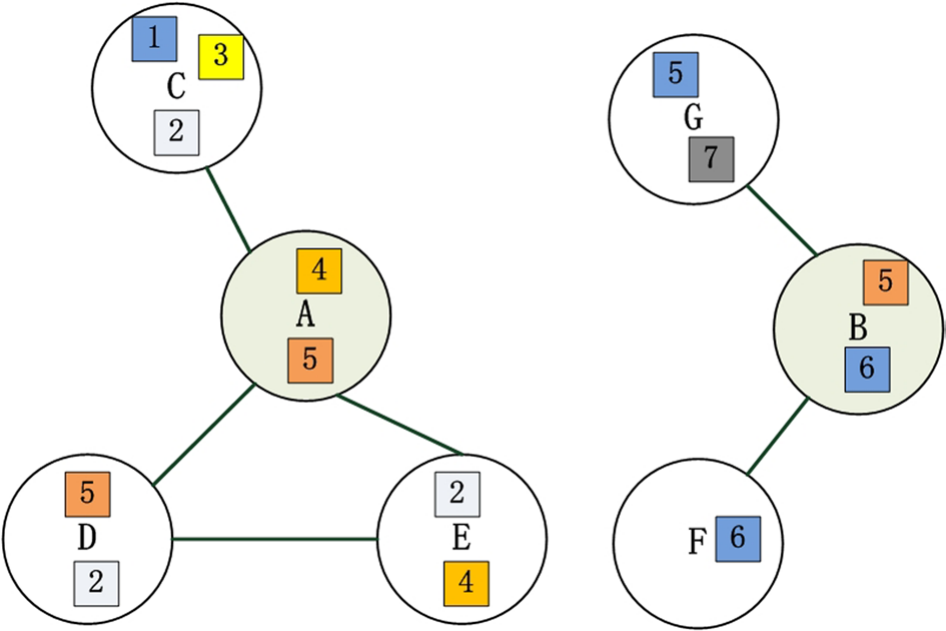
- Illustration of domain content similarity. The figure gives an example of the domain content similarity of protein A and B, in which rectangles in different colors are drawn to represent different types of domains. *D*_*A*_ = {1, 2, 3, 4, 5}, *D*_*B*_ = {5, 6, 7}, *DC* = *D*_*A*_ ∩ *D*_*B*_ = {5}

Figure 3 shows an example of calculating the domain content similarity of two proteins. Protein A and its four neighbors contain five different domains, while protein B involves three types of domains along with its two neighbors. The functional similarity of co-structure association between *p*_*i*_ and *p*_*j*_ is measured by the normalization processing of their domain content similarity, which is formally described as follows:

3 $$t(i,j,2) = \frac{{DS(p_{i} ,p_{j} ) - \mathop {\min }\limits_{1 \le i \le n,1 \le j \le n} (DS(p_{i} ,p_{j} ))}}{{\mathop {\max }\limits_{1 \le i \le n,1 \le j \le n} (DS(p_{i} ,p_{j} )){ - }\mathop {\min }\limits_{1 \le i \le n,1 \le j \le n} (DS(p_{i} ,p_{j} ))}}$$

Most cellular functions are carried out through interactions between multiple functional modules at various levels [26]. It is evident that the functional module or protein complex information is important for protein function prediction. If two proteins participate in the same module, they are likely to perform the same or similar functions. Firstly, we calculate the density score of experimental detection functional modules, which can be expressed as:

4 $$Score(M_{k} ) = \frac{{2 \times |E_{k} |}}{{|V_{k} | \times (|V_{k} | - 1)}}$$

where *E*_*k*_ and *V*_*k*_ denotes the set of physical interactions and proteins in the module *M*_*k*_, respectively. After getting the score of benchmark functional modules using Eq. (4), we can evaluate the reliability of co-module association between proteins. For any two proteins *p*_*i*_ and *p*_*j*_ in networks, their functional similarity based on co-module association is calculated as follows:

5 $$t(i,j,3) = \frac{{\left( {\sum\nolimits_{{k = 1,p_{i} \in M_{k} ,p_{j} \in M_{k} }}^{|M|} {Score(M_{k} )} } \right)^{2} }}{{\sum\nolimits_{{k = 1,p_{i} \in M_{k} }}^{|M|} {Score(M_{k} )} \times \sum\nolimits_{{k = 1,p_{j} \in M_{k} }}^{|M|} {Score(M_{k} )} }}.$$

### Random walk with restart on the tensor *T*

In this paper, multiple functional associations are introduced in the constructed tensor *T*. Therefore, the significance of proteins is taken into account in the iterative process as well as different types of interaction. Furthermore, our model is based on two hypotheses: proteins with high significance values connect to each other through significant interactions, and interactions with high significance values frequently are joined by many significant proteins. In this stage, our model performs iteration on the tensor *T* for a target protein to obtain functional association scores with other known proteins. Given a target protein *p*_*i*_, $X_{i} = [x_{i1} ,x_{i2} , \ldots ,x_{in} ]^{T} \in {\mathbb{R}}^{n}$ and $Y_{i} = [y_{i1} ,y_{i2} , \ldots ,y_{in} ]^{T} \in {\mathbb{R}}^{n}$ is the vector representing functional similar scores with known proteins and significance scores of different categories of interaction between proteins, respectively. We can thus extend the classical random walk with restart algorithm to the tensor model. Our method performs a two-step iteration operation to update significance scores of proteins and different types of interaction by:

6 $$\begin{aligned} X_{i}^{t + 1} & = \alpha \tilde{T}X_{i}^{t} Y_{i}^{t} + (1 - \alpha )X_{i}^{0} \\ Y_{i}^{t + 1} & = \tilde{T}^{\prime}X_{i}^{t} X_{i}^{t + 1} \\ \end{aligned}$$

where $\sum\nolimits_{j = 1}^{n} {x_{ij} } \, = \, 1$ and $\sum\nolimits_{k = 1}^{m} {y_{ik} } \, = \, 1$, $\tilde{T}$ and $\tilde{T}^{\prime}$ are got from the tensor *T* constructed in the first stage by normalizing so that entries in each row sum to 1, and they are calculated as follows:

7 $$\tilde{t}_{i,j,k} = \left\{ {\begin{array}{*{20}l} {\frac{{t_{i,j,k} }}{{\sum\nolimits_{i = 1}^{n} {t_{i,j,k} } }}} \hfill & {{\text{if}}\;\sum\nolimits_{i = 1}^{n} {t_{i,j,k} > 0} } \hfill \\ {1/n} \hfill & {{\text{otherwise}}} \hfill \\ \end{array} } \right. \,$$

8 $$\tilde{t}^{\prime}_{i,j,k} = \left\{ {\begin{array}{*{20}l} {\frac{{t_{i,j,k} }}{{\sum\nolimits_{k = 1}^{m} {t_{i,j,k} } }}} \hfill & {{\text{if}}\;\sum\nolimits_{k = 1}^{m} {t_{i,j,k} } > 0} \hfill \\ {1/m} \hfill & {{\text{otherwise}}} \hfill \\ \end{array} } \right.$$

The parameter $\alpha \in [0,1]$ is the probability of restart, and it balances between the iteration information and initial significant scores, which is set to 0.5 [27, 28]. Due to the low characteristic path length of the PPI network, nodes may not be able to return to their initial positions after the iterative process. While the random walk with restart model applied in our method can ensure that a seed node can iteratively move to a random neighbour with probability α or return to its original location with probability 1-α. It also guarantees the convergence of iteration on the tensor. In the Eq. (6),

$X_{i}^{0} = [x_{i1}^{0} ,x_{i2}^{0} , \ldots ,x_{in}^{0} ]^{T} \in {\mathbb{R}}^{n}$ is the vector of initial functional similar scores, and its element $X_{i,j}^{0}$ can be calculated as:

9 $$d_{ij} = \frac{{|D_{i} \cap D_{j} |}}{{\sqrt {|D_{i} | \times |D_{j} |} }} + \frac{{|C_{i} \cap C_{j} |}}{{\sqrt {|C_{i} | \times |C_{j} |} }}$$

10 $$X_{i,j}^{0} = d_{ij} /\sum\limits_{j = 1}^{n} {d_{ij} }$$

where *D*_*i*_ and *D*_*j*_ denotes the set of domains contained by protein *p*_*i*_ and *p*_*j*_, repectively. *C*_*i*_ and *C*_*j*_ represents the set of functional modules in which *p*_*i*_ and *p*_*j*_ is involved, respectively. Following iterations for all proteins, we obtain a functional similarity matrix *M*_*fs*_, which is formally described as follows:

11 $$M_{fs} = \left[ {\begin{array}{*{20}c} {x_{11} } & {x_{12} } & \cdots & {x_{1n} } \\ {x_{21} } & \ddots & {} & \vdots \\ \cdots & \cdots & \ddots & \vdots \\ {x_{n1} } & \cdots & \cdots & {x_{nn} } \\ \end{array} } \right]$$

The proof of convergence for the random walk on tensor algorithm is related to our previous work [29].

### Computational annotation of proteins with unknown functions

Benefiting from the iteration on the constructed tensor, we are able to discover more partners with similar functions to target proteins, which are ignored by PPI networks. These partners as well as the target protein interact with each other to carry out biological functions within multiple functional modules. Intuitively, members within the same functional module are often more densely connected than those across functional modules [30]. Unfortunately, some of these partners are pretenders who have closer connections to the outside of the module than to the inside. Therefore, those pretenders should be removed from the functional module. We introduce the concept of cohesiveness coefficient (CC) to assess whether a partner is false. Let *fs*^*in*^(*p*_*i*_) denote the total functional similarity score between all other proteins inside the functional module and the protein *p*_*i*_, and let *fs*.^*out*^(*p*_*i*_) denote the total functional similarity score between all proteins outside the functional module and the protein *p*_*i*_. The cohesiveness coefficient of *p*_*i*_ is then given by

12 $$CC(p_{i} ) = \frac{{fs^{in} (p_{i} )}}{{fs^{in} (p_{i} ) + fs^{out} (p_{i} )}}$$

where $fs^{in} (p_{i} ) = \sum\nolimits_{{p_{j} \in M}} {x_{ij} }$, $fs^{out} (p_{i} ) = \sum\nolimits_{{p_{k} \notin M}} {x_{ik} }$, and *M* is a set of partners that have same or similar functions as the target protein. Cohesiveness coefficient provides an efficient way to assess whether a functional module satisfies the properties of high cohesion and low coupling. A well-separated module consisted of many proteins with similar function has a high *fs*^*in*^ and a low *fs*^*out*^. For a partner with similar functions to the target protein, its *CC* value is less than or equal to1/3 [31] implies that it has more external functional similarity than internal functional similarity and should be removed from the partners group. After cohesiveness-filter processing using Eq. (11), we are able to get a functional similarity proteins set *FSP* = {*fsp*_1_, *fsp*_2_, …, *fsp*_*l*_}. Let *F* = {*f*_1_, *f*_2_, …, *f*_*m*_} be a list of distinct functions of proteins in *FSP*. We score and rank functions within *F* in descending order to annotate the target protein with top *K* of them. Given a function *f*_*i*_ for the target protein *p*_*t*_, its ranking score is calculated by the following formula:

13 $$RS(f_{i} ) = \sum\limits_{k = 1}^{l} {x_{{p_{t} ,fsp_{k} }} } , \, f_{i} \in GO(fsp_{k} )$$

where $x_{{p_{t} ,fsp_{k} }}$ is the functional similarity score between partner *fsp*_*k*_ and the target protein *p*_*t*_, *GO* (*fsp*_*k*_) is a set of functions belonging to *fsp*_*k*_. In this paper, the parameter *K* was assigned as the number of functions of the protein within *FSP*, which had the highest functional similarity score to the target protein.

## Results

### Experimental data

To estimate the performance of RWRT for protein function prediction, we perform computational analysis on our method as well as other six competing algorithms, such as NC, Zhang, DCS, DSCP, PON and *GrAPFI* on two benchmark datasets from yeast, including DIP [32] and BioGRID [33]. The DIP dataset and BioGRID dataset updated to February 5, 2017 and Oct.28, 2017, respectively. The DIP dataset consists of 4,912 proteins and 22,129 interactions among the proteins, and the BioGRID dataset consists of 4,113 proteins and 26,105 physical interactions. Self-interactions and repeated interactions are removed from the two benchmark datasets. The annotation data of proteins used for validation is downloaded from GO official website [34]. We primarily investigate and analyze the annotations in the Biological Process (BP) category in this manuscript. To avoid too special and too general, only those GO terms that annotate at least 10 and at most 200 proteins will be retained in our experiments [12]. The protein domain data is retrieved from the PFAM database [35], which involves 1064 and 1026 distinct domain types related to 2945 and 2566 proteins of the DIP dataset and BioGRID dataset, respectively. The experimental detection functional modules set comes from CYC2008 [36], which makes up of 408 modules referring to 1465 and 1600 proteins in DIP and BioGRID, respectively. Table 1 lists the detail of the two datasets.

**Table 1:** Basic information of the two PPI networks

Dataset	Proteins	Interactions	Annotated proteins
BioGRID	4113	26,105	2716
DIP	4912	22,129	2814

### View of the constructed functional similarity tensor *T*

**Fig. 4:**
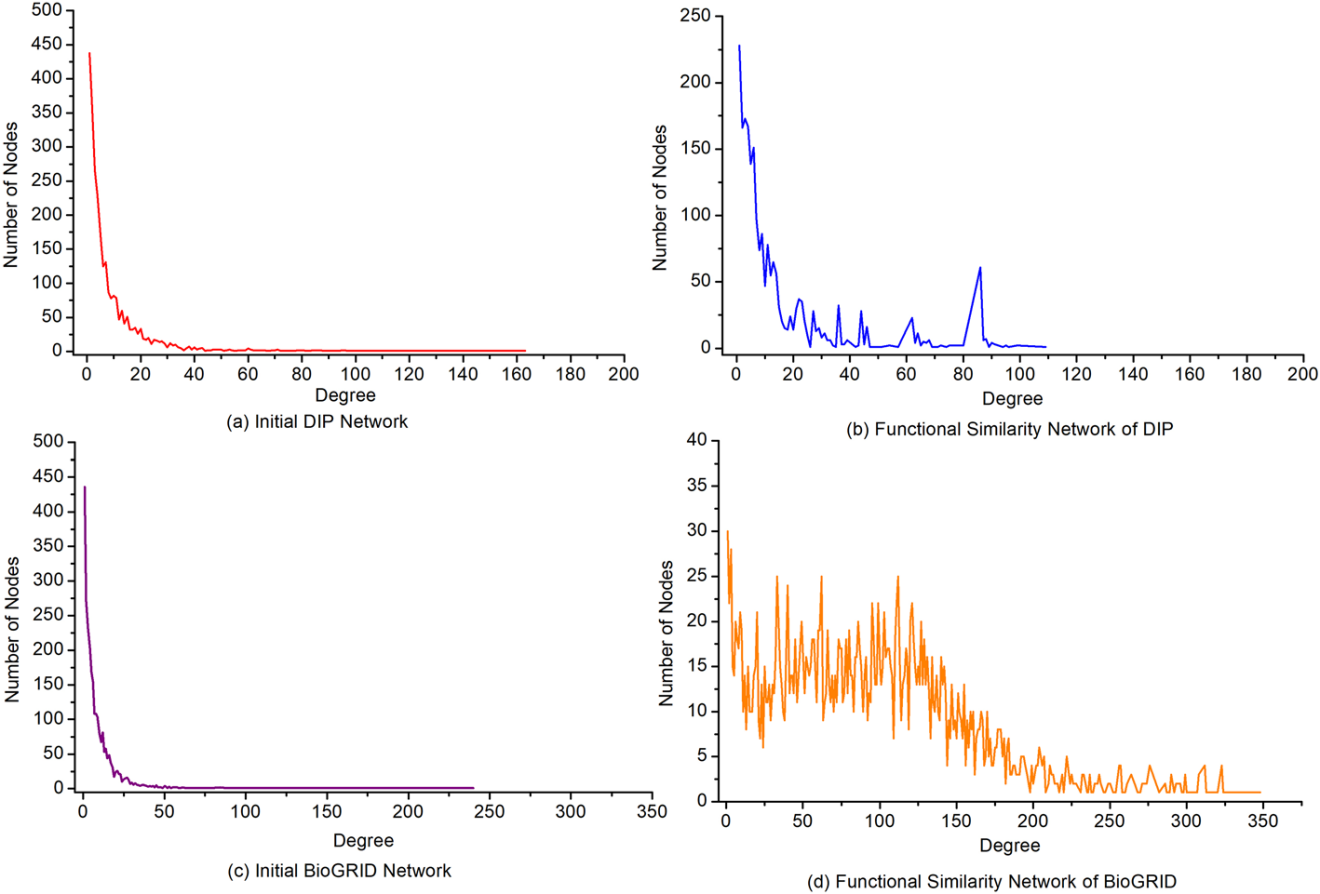
-The distribution of degree in four networks. This figure shows the distribution of degree in the two initial PPI networks and their corresponding functional similarity networks. **a** Initial DIP network, **b** Functional similarity network of DIP, **c** Initial BioGRID network, **d** Functional similarity network of BioGRID

For a better understanding of the behaviour of the proposed RWRT method, we provide descriptive statistics on the two PPI networks and their corresponding functional similarity networks, which are represented by functional similarity matrixes in Eq. (11). Table 2 lists the basic statistics of the four networks, such as average degree, clustering coefficient etc. Figure 4 depicts the distribution of degree in these four networks, respectively. Our statistics reveal higher cohesion and lower heterogeneity of the constructed functional similarity networks than their original network. So, it is reasonable to believe that the tensor-based random walk model is helpful to reduce the negative effect of false negative and improve the accuracy of prediction of protein functions.

**Table 2:** Statistics of initial networks and their corresponding functional similarity networks

Networks	Average degree	Clustering coefficient	Network density	Network heterogeneity
Initial DIP Network	9.396	0.153	0.004	1.385
Functional Similarity Network of DIP	21.509	0.859	0.017	1.101
Initial BioGRID Network	9.314	0.352	0.004	1.537
Functional Similarity Network of BioGRID	96.648	0.458	0.037	0.673

### Assessment criteria

To evaluate the effectiveness of RWRT and other six competing methods in protein function prediction, we apply cross validation to split proteins of PPI networks into the training set and the testing set using two strategies, which are leave-one-out cross validation and ten-fold cross validation. In one round of cross validation, the functions of each protein in the testing set are predicted according to the proteins in the training set. The validation process is repeated multiple times until each protein has an opportunity to become a member of the testing set. The final performances are evaluated by the average of all rounds. The process of function prediction using leave-one-out cross validation and ten-fold cross validation is described below.

To measure quality of the predicted functions by each method, we use two assessment criteria: AUROC (area under the receiver-operating curve) [37] and average F-score [38, 39]. AUROC is widely used in performance evaluation for protein function prediction. As the harmonic mean of Precision and Recall, F-score is another measure to evaluate the performance of a method synthetically. Precision is the fraction of predicted functions that are matched with known proteins while Recall is the fraction of known functions that are matched with predicted functions. In this study, true positive (TP), true negative (TN), false positive (FP) and false negative (FN) represents the number of matched predicted functions, matched known functions, incorrectly matched predicted functions and missing matched known functions, respectively.

### Leave-one-out cross-validation

**Fig. 5:**
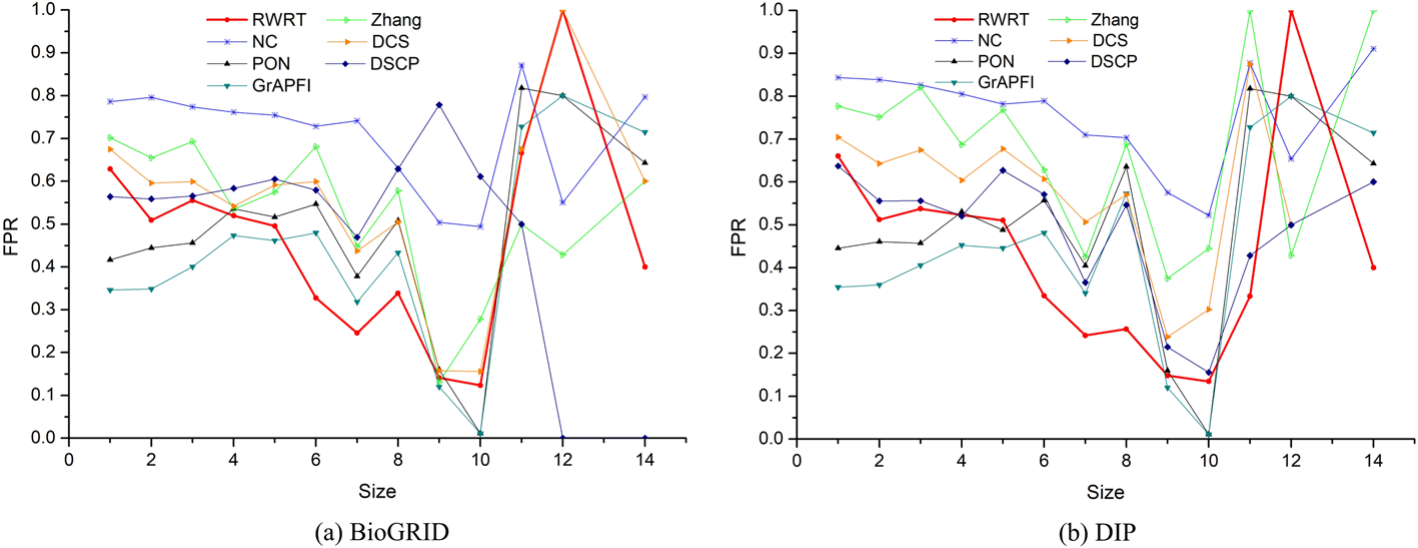
- The average FPR of seven methods according to different functional size of target proteins. The figure depicts the average false positive rate of our method and other competing methods fluctuate under different functional size of target proteins. Here functional size means the number of GO terms in each target protein. Size ranges from 1 to 14, except for 13. **a** Predicting results of seven methods on the BioGRID dataset. **b** Predicting results of seven methods on the DIP dataset

**Fig. 6:**
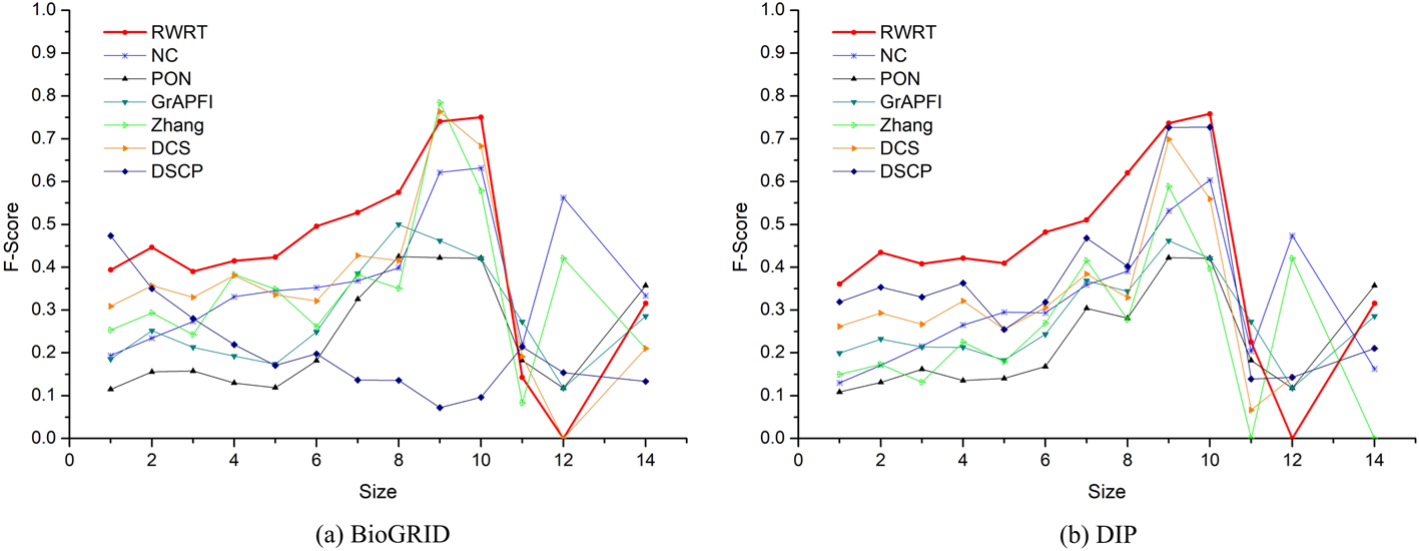
- The average F-Score of seven methods according to different functional size of target proteins. The figure shows the average F-Score of RWRT and other competing methods fluctuate under different functional size of target proteins. **a** Predicting results of seven methods on the BioGRID dataset. **b** Predicting results of seven methods on the DIP dataset

In our first set of evaluations, we apply leave-one-out cross validation to assess quality of the predicted functions predicted by RWRT, as well as other six competing methods: NC, Zhang, DCS, DSCP, PON and *GrAPFI*. The performance is averaged with only one protein into the testing set and rest of proteins used as the training set. We first evaluate the performance of RWRT and six competing methods on these target proteins by the average Precision, TPR (True Positive Rate), FPR (False Positive Rate) and F-Score. Table 3 lists the prediction results of RWRT and other competing methods. RWRT achieves the highest average Precision, TPR and F-Score values, and the lowest FPR values among the seven methods. Take the DIP dataset as an example, the average F-Score of RWRT is 107.46%, 135.59%, 41.84%, 18.47%, 218.32% and 93.06% higher than NC, Zhang, DCS, DSCP, PON and *GrAPFI*, respectively. For comprehensive performance comparison between RWRT and competing methods, we employ piecewise statistics of the predicted results according to functional size of target proteins. The results, shown in Figs. 5 and 6, suggest that these methods get different performance for different size of proteins, each with its own unique advantages. The performance of our RWRT has obvious advantages with size falls into [2, 8], while the prediction accuracy of DSCP on BioGRID data sets dropped sharply when size is in [6, 9]. Note that only four proteins have ten or more functional annotations. Therefore, the results of these methods in [10, 14] are not statistically significant and are not included in this analysis.

**Table 3:** The results of RWRT and six competing methods on the DIP and BioGRID dataset

Dataset	Methods	Precision	TPR	FPR	F-score
DIP	**RWRT**	**0.410**	**0.426**	**0.590**	**0.417**
	NC	0.126	0.491	0.831	0.201
	Zhang	0.176	0.179	0.761	0.177
	DCS	0.291	0.297	0.672	0.294
	DSCP	0.348	0.355	0.594	0.352
	PON	0.135	0.126	0.456	0.131
	*GrAPFI*	0.221	0.211	0.371	0.216
BioGRID	**RWRT**	**0.430**	**0.449**	**0.571**	**0.439**
	NC	0.172	0.633	0.780	0.270
	Zhang	0.292	0.301	0.666	0.297
	DCS	0.349	0.358	0.626	0.354
	DSCP	0.383	0.386	0.566	0.385
	PON	0.146	0.136	0.439	0.141
	*GrAPFI*	0.220	0.210	0.365	0.215

**Fig. 7:**
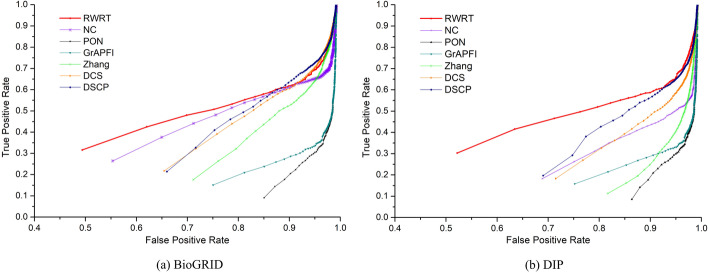
- ROC curves of seven methods using leave-one-out cross validation. The figure denotes the ROC (receiver-operating) curves of RWRT and other six competing methods (Zhang, DCS, DSCP, PON and *GrAPFI*) based on the average prediction performance over all testing proteins. The vertical and horizontal coordination of the ROC curves are the values of true positive rate and false positive rate, respectively. **a** ROC curves of seven methods on the BioGRID dataset. **b** ROC curves of various methods on the DIP dataset

To further investigate the performance of RWRT and six competing methods, we adopt the ROC curve, whose vertical and horizontal coordination are the values of TPR and FPR, respectively. Figure 7a and b depicts the ROC curve of seven methods on the BioGRID dataset and DIP dataset, respectively. For an intuitive evaluation of the performance of various methods, we calculate the area under all curves and list the results in Table 4. The AUROC of RWRT on BiosGRID is 18.43%, 102.36%, 51.18%, 47.70%, 576.32% and 283.58% higher than that of NC, Zhang, DCS, DSCP, PON and *GrAPFI*, respectively. As for the DIP dataset, the AUROC of RWRT increases by no less than 60% compared with other competing methods. Comparison results also reveal a phenomenon that the performance of these methods in the DIP dataset is significantly inferior to that of them on the BioGRID dataset. For NC, Zhang, DCS and DSCP, which mainly depend on neighbors, the gap is even more obvious. In our opinion, it is due to the fact that the DIP network is sparser than the BioGRID network. However, this does not appear to have much effect on the RWRT method. Experimental comparison results strongly prove the effectiveness and robustness of our method.

**Table 4:** AUROC of RWRT and other competing methods on the DIP and BioGRID dataset

Dataset	RWRT	NC	Zhang	DCS	DSCP	PON	*GrAPFI*
BioGRID	**0.257**	0.217	0.127	0.170	0.174	0.038	0.067
DIP	**0.237**	0.114	0.055	0.117	0.145	0.035	0.067

To analyze why RWRT obtains superior performance for the prediction of protein function, we investigate full matching, perfect matching, etc. between the benchmark set and the predicted set by the seven approaches. Table 5 lists matching results of RWRT and six competing methods on the two PPI networks. In Table 5, *OM* is the number of proteins that match at least one function, while *FM* is the number of proteins whose functions are fully matched and *ZM* is the number of proteins annotated by zero mismatching functions. *PM* is the number of proteins perfectly matching the known functions. In other words, a prediction has the same functions with the known functions matched with it. From Table 5, we can see that RWRT contains the second-biggest number of matched proteins (*OM*) and number of fully matched proteins (*FM*) after NC, while *ZM* and *PM* of our method is far higher than NC’s. The low precision of the NC method is mainly limited by its unweighted strategy, which is similar to that of the *GrAPFI* and PON method. Researches show that if the weight of an interaction reflects its reliability, then the weighted interactions should better represent the actual interaction network than the initial binary ones. RWRT archives the biggest number of perfect matching proteins (*PM*) and number of zero mismatching proteins (*ZM*), which is due in large part to the constructed tensor model for the integration of multi-omics data. In addition, predicted functions of RWRT, NC, PON and *GrAPFI* are derived from multiple functional neighbors, while that of Zhang, DCS and DSCP only come from the most similar protein. Our statistics show that nearly 68 percent of proteins have partners whose functions completely overlap, and more than 70 percent of these have only one function. Proteins interact with each other to form functional modules or protein complexes and perform useful cellular functions. Although some redundancy may be of biological importance, functional modules overlapping keep within a certain extent. So, we believe that the strategy of annotating target proteins with the functions of multiple proteins is sensible, which is especially favorable to proteins with large functional sizes.

**Table 5:** The matching results of RWRT and six competing methods on the DIP and BioGRID dataset

Dataset	Methods	*OM*	*FM*	*ZMM*	*PM*
DIP	**RWRT**	**1499**	**897**	**832**	**533**
	NC	1607	1121	85	76
	Zhang	661	371	352	244
	DCS	1069	634	608	429
	DSCP	1249	766	746	522
	PON	521	212	261	212
	*GrAPFI*	805	401	450	401
BioGRID	**RWRT**	**1562**	**896**	**809**	**536**
	NC	1945	1428	100	84
	Zhang	1071	591	557	371
	DCS	1244	737	698	485
	DSCP	1315	802	796	547
	PON	536	229	277	229
	*GrAPFI*	774	123	136	123

To assess the relative importance of each type of biological information on protein function prediction, we try to remove these multi-omics data respectively and run our RWRT method. Ablation results of RWRT on the DIP and BioGRID dataset are shown in Table 6. From Table 6 we can see that each interaction data source plays a different role in the prediction of protein function. The loss of functional module information has the greatest impact on the performance degradation of RWRT method, followed by that of domain context and PPI network topology.

**Table 6:** Ablation results of RWRT on the DIP and BioGRID dataset

Dataset	Conditions	Precision	TPR	FPR	F-score
DIP	PPIs removed	0.334	0.346	0.666	0.340
	Co-structure removed	0.308	0.304	0.692	0.306
	Co-module removed	0.237	0.228	0.763	0.232
BioGRID	PPIs removed	0.360	0.380	0.640	0.370
	Co-structure removed	0.331	0.340	0.669	0.335
	Co-module removed	0.315	0.336	0.685	0.325

In the RWRT method, we obtain a functional similarity network by performing iterative operations on the tensor model, which is formally described by Eq. (11). To verify the effectiveness of the tensor representation in fusing multi-omics data for protein function prediction, we run another version of the RWRT method named single-RWRT to annotate target proteins, in which the functional similar network is replaced by a single network. The single network *SN* is summarized by three types of physical or functional association involved in constructing the functional similarity tensor *T*. For a pair of proteins *p*_*i*_ and *p*_*j*_, the weight of edge (*p*_*i*_, *p*_*j*_) in *SN* is defined as:

14 $$SN(p_{i} ,p_{j} ) = a*t(i,j,1) + b*t(i,j,2) + (1 - a - b)*t(i,j,3)$$

where $a \in (0,1), \, b \in (0,1)$ and *a* + *b* < 1. *t*(*i*, *j*, 1), *t*(*i*, *j*, 2) and *t*(*i*, *j*, 3) is calculated in Eqs. (1), (3) and (5), respectively. Table 7 lists the comparison results between the single-RWRT and RWRT on the BioGRID dataset and DIP dataset. The optimal parameters of the single-RWRT are set according to the two dataset. Table 7 indicates that RWRT outperforms single-RWRT on two PPI networks obviously. At the same time, we also run the NC [1] method on the single network *SN*. The prediction results show that the recall (TPR) is close to 1, but the precision is very low. This is largely due to the fact that almost all known proteins are picked out as candidates to annotate the target protein. Integrating multiple biological data into a credible single network can indeed improve network connectivity and effectively eliminate false negatives in PPI networks, which leads to an increase in recall. However, it also inevitably introduces a lot of noise and reduces the precision of prediction. So, any increase in recall is more than offset by the accompanying increase in false positives [2]. The comparison results between the single-RWRT and RWRT strongly confirm the effectiveness of the tensor model.

**Table 7:** Comparison results between single-RWRT and RWRT on two datasets

Dataset	Methods	Precision	TPR	FPR	F-score
DIP	Single-RWRT	0.193	0.203	0.807	0.198
	RWRT	0.410	0.426	0.590	0.417
BioGRID	Single-RWRT	0.322	0.328	0.678	0.325
	RWRT	0.430	0.449	0.571	0.439

### Ten-fold cross validation

**Fig. 8:**
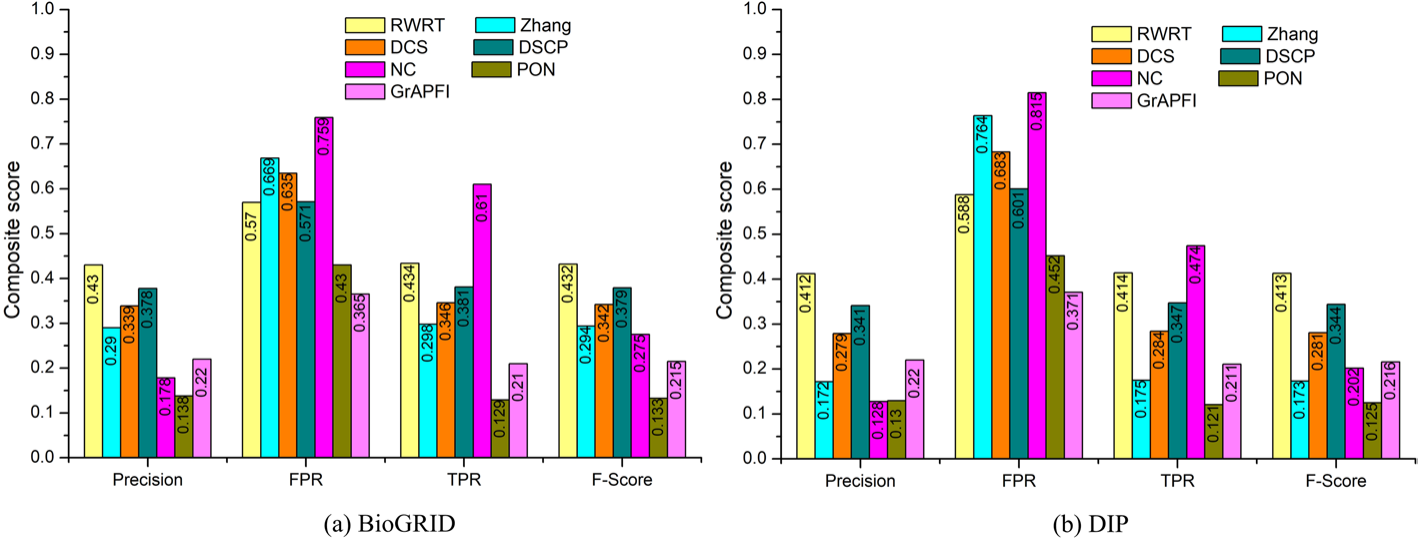
- The predicted results of various methods using ten-fold validation. Numbers of each bar are the values for each score, including average Precision, TPR (True Positive Rate), FPR (Fale Positive Rate) and F-score. **a** Results of seven methods on the BioGRID dataset. **b** Results of various methods on the DIP dataset

**Fig. 9:**
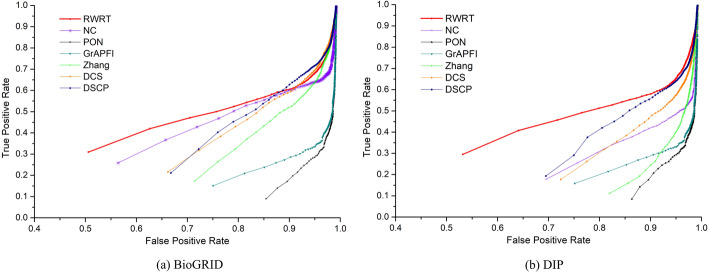
- ROC curves of seven methods using ten-fold cross validation. This Figure shows the ROC curves of RWRT and other six methods using ten-fold validation. The entire set of proteins is divided into ten equal sets randomly, nine of which are used for training and the remaining part is used for testing. The process is repeated 1000 times, each time using another testing set. **a** ROC curves of seven methods on the BioGRID dataset. **b** ROC curves of various methods on the DIP dataset

For comprehensive comparison between the novel method RWRT and the six other competing methods, we adopt the ten-fold cross validation to examine the stability of these methods on the training dataset. Proteins are randomly divided into ten subsets, a single subset is retained for the testing set, and the remaining nine subsets are used as the training set. The cross validation process is then repeated ten rounds, each of which uses different folds as the training and validation data. Ten results from the folds are then averaged to produce the final performance. We run functional annotation methods of RWRT as well as other six competing methods on the BioGRID and DIP network. Figure 8 presents the average Precision, TPR, FPR and F-score of seven methods on two datasets. Additionally, we draw ROC curves of all methods on the two PPI networks, which are illustrated in Fig. 9. The AUROC of RWRT on BiosGRID is 19.38%, 604.49%, 271.73%, 96.42%, 51.75% and 48.28% higher than that of NC, PON, *GrAPFI*, Zhang, DCS and DSCP, respectively. On the DIP dataset, AUROC of RWRT increases by 110.79%, 559.46%, 241.54%, 322.87%, 103.87% and 61.80%, respectively, compared to the above six methods. All of these experimental results indicate that RWRT still outperforms other six competing methods using other validation.

## Discussions

Accurate annotation of protein functions is still a big challenge for understanding life in the post-genomic era. In spite of the advances in computational methods for protein function prediction, it still fails to achieve satisfactory prediction accuracy plagued by the incompleteness and errors in the original PPI data. To overcome this problem, the integration of different types of biological data has become an important and popular strategy, which has led to the emergence of various interactions between proteins. Each type of biological data has its own property or correlation and can play a different role in prediction of protein functions. Inspired by it, we set up a multidimensional data model and describe it formally with the tensor. To get rid of constraints of the small-world and scale-free properties of PPI networks, we extend the bi-random walks algorithm to the tensor model. In this way, we can discover more potential proteins with similar functions to target proteins and improve the true positive rate of prediction. However, enlargement of the traversal range of similar functional neighbors will inevitably lead to the increase of noise. The NC method is a typical example to illustrate the fact. For this purpose, we run the functional module detection algorithm based on the concept of cohesiveness coefficient to filter out pretenders and reduce the false positive rate of prediction. We are of the opinion that there are both overlaps and differences in functional annotation between proteins. It is not appropriate to annotate a target protein with all the functions of the protein most similar to it.

## Conclusions

In this paper, we design a novel protein function prediction method named RWRT by applying a tensor-based bi-random walks model. The RWRT method constructs a functional similarity tensor depending on the original PPI network as well as multi-omics data firstly. And then, it extends the random walk with restart algorithm to the tensor by simulating a high-order Markov chain. After this phase, RWRT can discover more functional similarity partners ignored by original protein interactions data. However, it also inevitably introduces some spurious nodes. Therefore, a pretender-filtering procedure is applied to remove possible pretenders loosely connected to the target protein and finally generate predicted functions from the remaining functional partners. Experimental comparison results on two PPI networks indicate that RWRT performs significantly better than the state-of-the-art methods and the proposed model can provide more insights for future study in PPI networks.

## Supplementary Information

**Additional file 1**. Tensor-based random walk model. This file provides the derivation process of how to extend random walk model from two-dimensional matrix to the tensor model.

## Data Availability

Publicly available datasets were analyzed in this study. This data and the RWRT program can be found here: https://github.com/husaiccsu/RWRT.

## References

[1] Schwikowski B, Uetz P, Fields S (2000). A network of protein–protein interactions in yeast. Nat Biotechnol.

[2] Chua HN, Sung WK, Wong L (2006). Exploiting indirect neighbours and topological weight to predict protein function from protein–protein interactions. Bioinformatics.

[3] Moosavi S, Rahgozar M, Rahimi A (2013). Protein function prediction using neighbor relativity in protein–protein interaction network. Comput Biol Chem.

[4] Vazquez A, Flammini A, Maritan A (2003). Global protein function prediction from protein–protein interaction networks. Nat Biotechnol.

[5] Nabieva E, Jim K, Agarwal A (2005). Whole-proteome prediction of protein function via graph-theoretic analysis of interaction maps. Bioinformatics.

[6] Cao R, Cheng J (2016). Integrated protein function prediction by mining function associations, sequences, and protein–protein and gene–gene interaction networks. Methods.

[7] Liao B, Li Y, Jiang Y (2014). Using multi-instance hierarchical clustering learning system to predict yeast gene function. PLoS ONE.

[8] Liang S, Zheng D, Standley DM (2013). A novel function prediction approach using protein overlap networks. BMC Syst Biol.

[9] Sarker B, Rtichie D W, Aridhi S. Exploiting complex protein domain networks for protein function annotation. In: International conference on complex networks and their applications. Springer, Cham, p. 598–610; 2018.

[10] Wei P, Min L, Lu C (2017). Predicting protein functions by using unbalanced random walk algorithm on three biological networks. IEEE/ACM Trans Comput Biol Bioinf.

[11] Zhao B, Wang J, Li M (2016). A new method for predicting protein functions from dynamic weighted interactome networks. IEEE Trans Nanobiosci.

[12] Zhang S, Chen H, Liu K (2009). Inferring protein function by domain context similarities in protein–protein interaction networks. BMC Bioinform.

[13] Peng W, Wang J, Cai J (2014). Improving protein function prediction using domain and protein complexes in PPI networks. BMC Syst Biol.

[14] Rehman H U, Benso A, Di Carlo S, et al. Combining homolog and motif similarity data with Gene Ontology relationships for protein function prediction. In: 2012 IEEE international conference on bioinformatics and biomedicine (BIBM). IEEE, p. 1–4; 2012.

[15] Piovesan D, Giollo M, Leonardi E (2015). INGA: protein function prediction combining interaction networks, domain assignments and sequence similarity. Nucleic Acids Res.

[16] Piovesan D, Tosatto SCE (2019). INGA 2.0: improving protein function prediction for the dark proteome. Nucleic Acids Res.

[17] O’Meara MJ, Ballouz S, Shoichet BK (2016). Ligand similarity complements sequence, physical interaction, and co-expression for gene function prediction. PLoS ONE.

[18] Makrodimitris S, Reinders MJT, Van Ham RCHJ (2020). Metric learning on expression data for gene function prediction. Bioinformatics.

[19] Gligorijević V, Renfrew PD, Kosciolek T (2021). Structure-based protein function prediction using graph convolutional networks. Nat Commun.

[20] Martiniano HFMC, Asif M, Vicente AM, et al. Network propagation-based semi-supervised identification of genes associated with autism spectrum disorder. In: International meeting on computational intelligence methods for bioinformatics and biostatistics. Springer, Cham, p. 239–248; 2018.

[21] Zhao BH, Zhao YL, Zhang XX (2019). An iteration method for identifying yeast essential proteins from heterogeneous network. BMC Bioinform.

[22] Zhang W, Ma J, Ideker T (2018). Classifying tumors by supervised network propagation. Bioinformatics.

[23] Novoa-del-Toro EM, Mezura-Montes E, Vignes M (2021). A multi-objective genetic algorithm to find active modules in multiplex biological networks. PLoS Comput Biol.

[24] Wang X, Yang LT, Kuang L (2019). A tensor-based big-data-driven routing recommendation approach for heterogeneous networks. IEEE Netw.

[25] Forslund K, Sonnhammer ELL (2008). Predicting protein function from domain content. Bioinformatics.

[26] Li J, Zhao PX (2016). Mining functional modules in heterogeneous biological networks using multiplex PageRank approach. Front Plant Sci.

[27] Taehyun H, Hugues S, Tian Z (2014). Robust and efficient identification of biomarkers by classifying features on graphs. Bioinformatics.

[28] Vanunu O, Magger O, Ruppin E (2010). Associating genes and protein complexes with disease via network propagation. PLoS Comput Biol.

[29] Zhao B, Zhang Z, Jiang M (2020). NPF:network propagation for protein function prediction. BMC Bioinform.

[30] Hartwell L, Hopfield J, Leibler S, Murray AW (1999). From molecular to modular cell biology. Nature.

[31] Nepusz T, Yu H, Paccanaro A (2012). Detecting overlapping protein complexes in protein–protein interaction networks. Nat Methods.

[32] Xenarios I, Salwinski L, Duan XJ (2002). DIP, the Database of Interacting Proteins: a research tool for studying cellular networks of protein interactions. Nucleic Acids Res.

[33] Oughtred R, Stark C, Breitkreutz BJ (2019). The BioGRID interaction database: 2019 update. Nucleic Acids Res.

[34] Ashburner M, Ball CA, Blake JA (2000). Gene Ontology: tool for the unification of biology. Nat Genet.

[35] Bateman A, Coin L, Durbin R (2004). The Pfam protein families database. Nucleic Acids Res.

[36] Pu S, Wong J, Turner B (2009). Up-to-date catalogues of yeast protein complexes. Nucleic Acids Res.

[37] Peng W, Tang Q, Dai W (2022). Improving cancer driver gene identification using multi-task learning on graph convolutional network. Brief Bioinform.

[38] Peng W, Yi S, Dai W (2021). Identifying and ranking potential cancer drivers using representation learning on attributed network. Methods.

[39] Song J, Peng W, Wang F (2019). An Entropy-based method for identifying mutual exclusive driver genes in cancer. IEEE/ACM Trans Comput Biol Bioinf.

