# Supplementary material for: A tensor-based bi-random walks model for protein function prediction
Source: BMC Bioinformatics. 2022 May 30;23:199. doi: 10.1186/s12859-022-04747-2 (PMC9150346; doi:10.1186/s12859-022-04747-2)
Supplement: Supplementary file 1 — Additional file 1. Tensor-based random walk model. This file provides the derivation process of how to extend random walk model from two-dimensional matrix to the tensor model. [file 12859_2022_4747_MOESM1_ESM.pdf]

THEOREM 1. Given a tensor T as well as the vector $X = [x_1, x_2, \ldots, x_n]^T \in \mathbb{R}^n$ and $Y = [y_1, y_2, \ldots, y_n]^T \in \mathbb{R}^n$, which denotes important scores of proteins and different categories of interactions between proteins, respectively. The number of iteration depends on parameters α

Proof. We formally described the relationships between X and Y based on the tensor T using the following equation:

$$X = f(T, X, Y), Y = g(T, X) \tag{1}$$

The most critical task for us was to design reasonable functions f and g and to calculate X and Y respectively. We now propose the idea to define a higher-order Markov chain by normalizing the tensor. This leads to two probability transition tensors $T^{(1)} = (t^{(1)}{}_{ijk}) \in \mathbb{R}^{n\times n\times l}$ and $T^{(2)} = (t^{(2)}{}_{ijk}) \in \mathbb{R}^{n\times n\times l}$ that are calculated as follow:

$$t^{(1)}_{i,j,k} = \begin{cases} \dfrac{t_{i,j,k}}{\sum_{i=1}^{n} t_{i,j,k}} & if \sum_{i=1}^{n} t_{i,j,k} > 0 \\ 1/n & otherwise \end{cases} \tag{2}$$

$$t^{(2)}_{i,j,k} = \begin{cases} \dfrac{t_{i,j,k}}{\sum_{k=1}^{m} t_{i,j,k}} & if \sum_{k=1}^{m} t_{i,j,k} > 0 \\ 1/m & otherwise \end{cases} \tag{3}$$

We can then easily obtain the following formulas:

$$0 \le t^{(1)}_{i,j,k} \le 1, \sum_{i=1}^{n} t^{(1)}_{i,j,k} = 1 \tag{4}$$

$$0 \le t^{(2)}_{i,j,k} \le 1, \sum_{k=1}^{m} t^{(2)}_{i,j,k} = 1 \tag{5}$$

Equation (2) and Equation (3) can be interpreted as the transition probabilities of two third-order Markov chains $(X_t)_{t\in\mathbb{N}}$ and $(Y_t)_{t\in\mathbb{N}}$, respectively.

$$t^{(1)}_{i,j,k} = P[X_t = i \mid X_{t-1} = j, Y_t = k] \tag{6}$$

$$t^{(2)}_{i,j,k} = P[Y_t = k \mid X_t = i, X_{t-1} = j] \tag{7}$$

If the last state was the i-th node, then the next state is the j-th node through the k-th type of interaction with probability $t^{(1)}_{i,j,k}$. Similarly, $t^{(2)}_{i,j,k}$ can be considered as the probability of selecting the

k-th type of interaction from the *j*-th node to the *i*-th node. For the calculation of the random variables *X* and *Y*, the above two equations are deduced according to the total probability formula as follows:

$$P[X_t = i] = \sum_{j=1}^{n}\sum_{k=1}^{m} t_{i,j,k}^{(1)} \times P[X_{t-1} = j, Y_t = k] \tag{8}$$

$$P[Y_t = k] = \sum_{i=1}^{n}\sum_{j=1}^{n} t_{i,j,k}^{(2)} \times P[X_t = i, X_{t-1} = j] \tag{9}$$

$P[X_{t-1} = j, Y_t = k]$ represents the joint probability distribution of X_{t-1} and Y_t, and $P[X_t = i, X_{t-1} = j]$ denotes the joint probability distribution of X_{t-1} and X_t. Considering the steady state of the Markov chain, we can obtain the follow formulas:

$$x_i = \lim_{t\to\infty} P[X_t = i], \quad (1 \le i \le n) \tag{10}$$

$$y_k = \lim_{t\to\infty} P[Y_t = k], \quad (1 \le k \le m) \tag{11}$$

It is very difficult to calculate *X* and *Y* due to their coupling to each other and the observation that they contain two joint probability distributions in Equations (8) and (9). In this study, we assumed that the random variables *X* and *Y* were completely independent of each other. Thereafter, we could obtain these following formulas:

$$P[X_{t-1} = j, Y_t = k] = P[X_{t-1} = j] P[Y_t = k] \tag{12}$$

$$P[X_t = i, X_{t-1} = j] = P[X_t = i] P[X_{t-1} = j] \tag{13}$$

Based on the above assumption and the fact that *t* continues to infinity, Equations (10) and (11) could be deduced as:

$$x_i = \sum_{j=1}^{n}\sum_{k=1}^{m} t_{i,j,k}^{(1)} x_j y_k, \ i = 1, 2, \ldots n \tag{14}$$

$$y_k = \sum_{i=1}^{n}\sum_{j=1}^{n} t_{i,j,k}^{(2)} x_i x_j, \ k = 1, 2, \ldots m \tag{15}$$

Based on this, we designed the proper solutions for the functions *f* and *g*. Therefore, the random walk with restart algorithm in the multiplex biological network case could be described as follows:

$$X_t = \alpha \times T^{(1)} \times X_{t-1} Y_{t-1} + (1-\alpha) \times X_0 \tag{16}$$

$$Y_t = T^{(2)} \times X_t^{\,2} \tag{17}$$

The restart vector, X_0, represents the initial probability distribution. α is the restart probability.
